# The Effect of Chlorhexidine Disinfectant Gels with Anti-Discoloration Systems on Color and Mechanical Properties of PMMA Resin for Dental Applications

**DOI:** 10.3390/polym13111800

**Published:** 2021-05-29

**Authors:** Zbigniew Raszewski, Danuta Nowakowska, Włodzimierz Więckiewicz, Agnieszka Nowakowska-Toporowska

**Affiliations:** 1 Research Assistant, SpofaDental Kerr Company, 506-01 Jičin, Czech Republic; zbigniew.raszewski@kavokerr.com; 2 Department of Prosthodontics, Wroclaw Medical University, 50-425 Wroclaw, Poland; danuta.nowakowska@umed.wroc.pl (D.N.); wlodzimierz.wieckiewicz@umed.wroc.pl (W.W.)

**Keywords:** ADS, poly(methyl methacrylate), color, chlorhexidine, local anti-infective agents

## Abstract

Chlorhexidine (CHX)-based dental hygiene products are widely used by dental patients. As these products may have long-term contact with denture poly(methyl methacrylate) (PMMA) resin, anti-discoloration systems (ADSs) were included in them to prevent discoloration of the natural teeth and dental materials. **Purpose:** The aim of this study was to evaluate the effect of two newly designed CHX-containing gels with ADSs and two commercial products with ADSs (Curasept 0.5% and Curasept 1%) in preventing staining and to analyze the mechanical properties of heat-curing PMMA denture base resin. **Materials and methods:** Twenty-five discs (five for each test group) of PMMA dental resin with a thickness of 1 mm and a diameter of 20 mm were polymerized according to the manufacturer’s instructions and stored in distillate water at a temperature of 37 °C. The surface of the specimens was covered with two commercially available gels—Curasept 1% and Curasept 0.5%, or two experimental gel formulations containing 1% CHX. PMMA specimens stored in distilled water were used as control. The initial values of color and Brinell hardness of the specimens were measured immediately after specimen preparation. The changes in color and Brinell hardness, as well as water sorption, and solubility of the specimens were measured after one year of conditioning. Statistical analysis of the obtained data was performed using one-way analysis of variance and Dunn–Bonferroni post hoc tests. **Results**: In the group of specimens covered with gel 1 with citric acid or Curasept 0.5%, the color change was clinically acceptable (ΔE* < 2.7). In the specimens stored in contact with gel 2 with polyvinylpyrrolidone (PVP) and Curasept 1%, the ΔE* values were 3.6 and 3.67, respectively. In the control group, the level of hardness decreased significantly from 150 to 140 during the experiment. In addition, a statistically significant decrease in hardness was observed in specimens stored with Curasept 1% and gel 2 with PVP. Specimens stored in contact with Curasept 0.5% and gel 1 with citric acid also showed a lower hardness, but the change was not statistically significant. The sorption of all the groups of PMMA specimens ranged from 22.83 to 24.47 µg/mm^3^, with no significant differences found between them. All the PMMA specimens stored in contact with the tested CHX gels exhibited a significantly higher solubility (6.84 ± 7.91 µg/mm^3^) compared to the control group (6.74 µg/mm^3^), with the highest solubility noted for specimens stored with Curasept 1%. **Conclusions**: The results showed that CHX used in the gel form with ADSs at a concentration of 0.5% and the experimental gel containing 1% CHX with citric acid caused limited changes to the color and mechanical properties of the PMMA denture base resin during the study period. These gels may be safely used by dental patients for oral hygiene regimen even for prolonged periods of time. ADSs contained in these gels seem to be effective in preventing CHX discoloration.

## 1. Introduction

Patients wearing acrylic removable partial and total dentures often suffer from mucosal irritation caused by prosthetic appliances, and may also be exposed to fungal and bacterial infections. Additionally, the good esthetic appearance of dentures after several years of service and adequate mechanical properties of poly(methyl methacrylate) (PMMA) resin are the key factors determining the success of prosthetic treatment. The condition of the mucosa is influenced by the following: health of the denture wearer; presence of systemic diseases, such as diabetes, vitamin deficiencies, or hormonal disturbances; medicaments; diet; smoking and alcohol use; quantity and quality of saliva; and frequent use of denture adhesives [1]. A reduced flow of saliva, accompanied by mucosal sensitivity, may increase the frequency of stomatopathies. An additional factor that contributes to oral mucosa irritation is the difficulty of maintaining good oral and denture hygiene, which is especially observed among geriatric patients, leading to increased dental and denture plaques in this group. According to the literature, poor oral hygiene may be a risk factor associated with the development of aspiration pneumonia because of the increased presence of respiratory pathogens in the oral cavity [2]. To prevent this infection, the oral cavity should be protected for long term from bacterial and fungal colonization with the use of disinfecting products. Also, recent studies indicate that the use of disinfectant substances such as CHX, H_2_O_2_, cetylpyridinium chloride, and iodopovidone may play an important role in SARS-CoV-2 transmission prevention as oral tissues and saliva may be a virus reservoir [3,4,5]. A majority of currently used oral disinfectant products are based on chlorhexidine digluconate, quaternary amines, alkaline peroxides, alkaline hypochlorites, diluted acids, and enzymes [6,7,8]. However, these substances may affect the color of the dental acrylic devices as well as the mechanical properties of the PMMA resin [9]. PMMA is widely used in dentistry for a variety of applications in prosthodontics and orthodontics. This material is used for manufacturing of denture teeth and bases, palatal obturators, fixed interim restorations, occlusal splints, bite guards, orthodontic devices, and retainers as well as their repair procedures. Additionally, in fabrication of individual trays and surgical guides for implantology. PMMA may be processed in pouring techniques such as heat, cold, and light cured resins; injection molding; CAD/CAM systems; and three-dimensional (3D) printing [10,11,12].

CHX is one of the most commonly used drugs in dental therapy. It is very effective against different kinds of microorganisms and exhibits a broad-spectrum biocidal effect against both Gram-positive end Gram-negative bacteria. It is also active against yeasts as well as some dermatophytes and lipophilic viruses. At low concentrations, CHX shows a bacteriostatic effect, while at high concentrations it acts as a bactericide causing bacterial cell death by cytolysis [13]. In dentistry, it is used in the form of oral gels, mouth rinses, tooth mousses, or dentifrices, and as a component of dental floss or prophylactic and medicinal products. The approved concentration of active CHX ranges from 0.1% for daily use up to 3% for special cases. The use of 0.2% CHX significantly reduces oral colonization and is recommended as an easier and more cost-effective alternative to a traditional hygiene protocol for geriatric edentulous patients [2]. Numerous studies have confirmed that CHX is beneficial in reducing the accumulation of dental and denture plaques, tooth caries, gingivitis, periodontitis, and alveolar osteitis. However, CHX also exhibits cytotoxic activity on human cells, and can cause colorization of teeth and fillings. The activity of CHX depends on the pH of the environment and the presence of organic substances [13]. Brookes et al. concluded that, to ensure safety, the use of CHX in dentistry and oral healthcare should be oral-disease-specific as it exhibits different mechanisms of action on different microbes [14]. A study of Opstrump et al. also reported that the use of CHX is associated with an increased risk of allergy [15].

The main drawback of CHX-based medicaments used in dentistry is that they may cause taste disorders, bitterness, and extrinsic staining of natural dentition as well as a wide range of dental materials [16]. This is related to the local precipitation reaction occurring between the cationic CHX molecule bound to teeth or dental materials and the chromogens found in the saliva during the intake of food and beverages [17]. Several publications have documented the influence of CHX disinfectants on the color and mechanical properties of acrylic resins [18,19,20,21,22,23,24,25,26,27,28,29]. In general, alteration of color can have a negative impact on patient satisfaction and indicate the aging of the material. This is more pronounced in the case of self-curing acrylic resins, the color of which can easily change. This can be explained by the amine redox system, as well as by other factors such as the dissolution of ingredients, degradation of intrinsic pigments, and the chemical composition of resins [2]. Commercial products based on CHX containing anti-discoloration systems (ADSs) were introduced to overcome this problem. Some studies confirmed that the presence of ADSs in mouth rinses did not reduce the antiplaque action of CHX, which suggests that their addition to CHX-based oral hygiene products can be beneficial [30,31,32,33,34]. However, there is no consensus on the efficiency of ADSs in staining prevention, and the effect of long-term contact of CHX-based products containing ADSs with dental materials also needs to be elucidated.

The present study tested the anti-staining effect of two newly developed experimental CHX-based gel formulations with ADSs, as well as that of two commercial CHX-based gels with ADSs, to determine if their long-term use has an effect on the color of heat-curing PMMA resin. In addition, the effect of these gels on the Brinell hardness, sorption, and solubility of the denture material was evaluated. The null hypothesis of this study was that CHX-based gels with ADSs do not cause any color change or affect the mechanical properties of heat-cured PMMA resin.

## 2. Materials and Methods

### 2.1. PMMA Specimen Preparation

Heat-curing acrylic resin Spofacryl (SpofaDental, Jilčin, Czech Republic) was polymerized according to the manufacturer’s instructions in disc-shaped metal molds with a thickness of 1 mm and a diameter of 20 mm. The specimens were covered by a polyester foil and two metal slabs on each side. Initial polymerization was carried out for 30 min at a temperature of 60 °C and subsequently for 1 h at 100 °C. After polymerization, the specimens were removed from the forms and their surface was polished with a pumice and polishing paste (Ivoclar Vivadent, Schaan, Lichtenstain) for 5 min to obtain a smooth surface mimicking the polished part of the denture base. A total of 25 samples were prepared for the study. The prepared discs were divided into five groups, with five specimens in each.

### 2.2. Specimen Conditioning

The specimens were covered on one side with a disinfectant gel (0.5 g) and placed in glass containers containing distillate water at 37 °C. The CHX gels with ADSs used in the study and their composition are presented in Table 1. During the experiment, the discs were removed from the water bath every 7 d, washed with distillate water, and a new gel layer was placed on their surface. The distilled water in the glass containers was also changed. This process was carried out for 12 months. For the first two groups, experimental gels with 1% CHX were prepared according to the experimental formulation proposed by the authors of this study [20]. The third group of specimens was covered with Curasept 0.5% CHX gel, and the fourth group with Curasept 1% CHX gel. As a control group, five acrylic discs stored in distillate water at 37 °C were used for reference. At the end of the testing period, all the specimens were washed under tap water and dried with paper towels.

### 2.3. Color Evaluation

The initial color of the specimens was assessed immediately after specimen preparation using X Rite Spectrophotometer (X Rite Incorporated, Grand Rapids, MI, USA). The spectrophotometric device was calibrated using a white reference tile corresponding to the *L* (lightness) parameter. A measurement cleft of 4 mm was maintained during the measurements. The L, a, b scale was reduced for the values of surface smoothness (Specular Included or SPIN). The initial color of the specimens was noted as L_1_, a_1_, b_1_. Subsequently, the second measurement was carried out after the specimen conditioning period and the new values of color coordinates were noted as L_2_, a_2_, b_2_. Finally, the value of color change (ΔE*) was calculated using the formula [35]

(1) $$\Delta E^{*}=\sqrt{\left[{\left(L_{2}-L_{1}\right)}^{2}+{\left(a_{2}-a_{1}\right)}^{2}+{\left(b_{2}-b_{1}\right)}^{2}\right]}$$

where ΔE* is the change in the position of a point in the three-dimensional CIE L*a*b* space [36];

L is the axis lightness from black (0) to white (100);

a is the axis from red (−150) to green (100); and

b is the axis from yellow (−100) to blue (150).

### 2.4. Brinell Hardness Evaluation

The initial Brinell hardness of the specimens was assessed using a digital hardness tester (WPM, Leipzig, Germany), applying a load of 612 N for 120 s. The hardness of each specimen was tested two times, at the center and on the side, and the average value was calculated for all the groups. After the conditioning period, hardness was assessed again for all the tested groups of specimens.

### 2.5. Sorption and Solubility Evaluation

After preparation, all the specimens were weighted in Preciosa 501 analytical balance (Preciosa Stainberg System, Germany). The initial weight of the specimens was marked as M_1_ (μg). After the conditioning period, their weight was assessed again and marked as M_2_. Then, the specimens were placed into a desiccator under silica drying gel. Finally, the mass of the specimens after desiccation was measured and marked as M_1_. The water sorption and solubility of acrylic resin were calculated using the equation based on ISO 20795-1:2013 [37]

(2) $$\mathrm{Sorption}\;\left[\mu g/{\mathrm{mm}}^{3}\right]=\frac{M_{2}-M_{1}}{\mathrm{specimen}\;\mathrm{volume}}$$

(3) $$\mathrm{Solubility}\;\left[\mu g/{\mathrm{mm}}^{3}\right]=\frac{M_{1}-M_{3}}{\mathrm{specimen}\;\mathrm{volume}}$$

### 2.6. Statistical Analysis

Statistical analysis was performed with one-way analysis of variance for independent variables followed by Dunn–Bonferroni post hoc tests. Differences with *p* ≤ 0.05 were considered statistically significant.

## 3. Results

**Figure 1:**
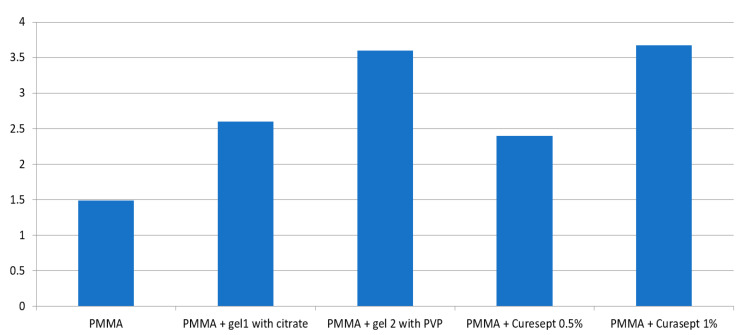
Color change (ΔE*) of the specimens after 1 year of conditioning with CHX gels.

The values of color change determined for the groups are presented in Table 2 and Figure 1. The ΔE* value of the control group (1.49) was significantly lower compared to the PMMA specimens stored in contact with all the tested CHX gels. Curasept 0.5% caused the smallest color change, followed by gel 1 with citric acid, while the highest color change was observed for gel 2 with PVP and Curasept 1%.

**Figure 2:**
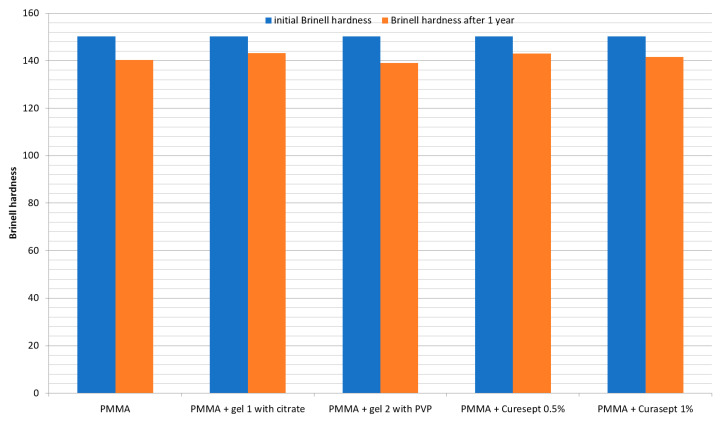
Initial and final Brinell hardness of the specimens after 1 year of conditioning.

The Brinell hardness values of the groups are presented in Table 3 and Figure 2. The hardness level of the control group was found to be decreased significantly (from 150 to 140) during the 1 year of storage in distilled water. Similarly, a statistically significant decrease in hardness was observed for the specimens stored with Curasept 1% and gel 2 with PVP. The PMMA specimens stored in contact with Curasept 0.5% and gel 1 with citric acid also showed lower hardness after the conditioning period, but the change was not statistically significant. The highest decrease in hardness was noted for the specimens stored with gel 2 with PVP (139.1) compared to the specimens stored with other tested gels.

**Figure 3:**
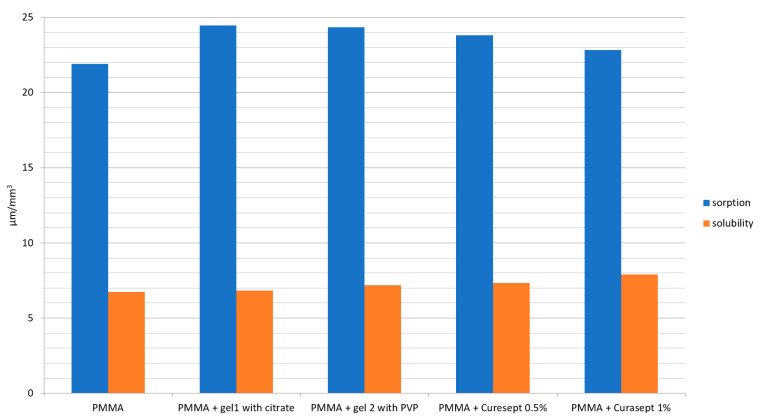
Sorption and solubility of the specimens after 1 year of conditioning.

Table 4 and Figure 3 present the sorption and solubility values of the specimens. No significant differences were noted between the sorption levels of the control group and the PMMA specimens stored in contact with the tested gels. The sorption of all the PMMA specimens ranged from 22.83 to 24.47 µg/mm^3^. On the other hand, all the PMMA specimens stored in contact with the tested CHX gels had a significantly higher solubility, ranging from 6.84 to 7.91 µg/mm^3^, while the control group had a solubility of 6.74 µg/mm^3^. The highest solubility was noted for the specimens stored with Curasept 1%.

## 4. Discussion

The null hypothesis assumed for this study was rejected. It was found that long-term contact with CHX gels containing ADSs influenced the color and, to some extent, the mechanical properties of PMMA resin. This effect was less pronounced in the case of product containing a lower concentration of CHX as well as for gel 1 with citric acid.

Spectrophotometry is an objective method for the assessment of color change based on the amount of light reflected by the specimen. This, in turn, facilitates the determination of ΔE*, which is the change in the position of a point in the three-dimensional CIE L*a*b* space [36]. The color difference thresholds for this scale were determined by Pavarina et al. [38]. The perceptibility threshold is ΔE* 1.22, while the acceptability threshold is ΔE* 2.66. In this study, the color change noted for the control group of specimens was slightly higher than the perceptibility threshold (ΔE* 1.49). In the case of specimens stored with gel 1 with citric acid and Curasept 0.5%, the color change was clinically acceptable. However, in specimens stored with gel 2 with PVP and Curasept 1%, the color difference exceeded the acceptability threshold. The color of these two groups of specimens changed toward yellow and was less transparent.

Patients using removable dentures often use different types of disinfectant gels. These products can contain CHX in their composition, to prevent various types of denture-associated stomatopathies [6,7,8,9,10,11,12,13,14,15,16,17,18,19,20]. Sometimes, these drugs may be used for a prolonged time, lasting for several weeks. The effects of rinses containing CHX were also widely discussed in other studies [6,7,8,9,10,11,12,13,14,15,16,17,18,19,20,25,26]. Patel et al. observed that CHX may affect the color of provisional crowns and bridges made with acrylic resin [18]. Moffa et al. tested hard relining PMMA material, Tokuyama Rebase, in contact with 2% CHX, in a group of patients for six months [7]. The color change was determined as 5.75 for the CHX group, which was higher than the values obtained in this study even though CHX was only used for a shorter period than that applied in the denture disinfection protocol. This finding was also confirmed in the present work, in which long-lasting contact of a CHX gel with acrylic material was found to cause color alteration. The color change can be more pronounced with the use of CHX at higher concentrations and without an ADS. By contrast, Drăghici et al. did not observe any large changes in the color of acrylic teeth treated with CHX for a period of 7 days [8]. This difference in results is probably due to the fact that the resin was in contact with CHX for a relatively short time. The second fundamental reason is that the test used a mixture of 0.2% CHX. In the case of CHX used in the gel form, the concentration was higher ranging from 0.5% to 1% and the contact period with the surface of the acrylic resin was longer.

The 1-year storage in distilled water significantly decreased the hardness of PMMA resin in both the control group and groups tested with gel 2 with PVP and Curasept 1%. This is probably due to the fact that water, which is the main component of the gel, penetrates mainly into the resin matrix. It was noted that 1-year contact with Curasept 0.5% and gel 1 with citric acid caused no statistically significant change in the hardness levels of the PMMA resin. Specimens stored with Curasept 1% showed a slight decrease in hardness. A similar effect on the hardness of PMMA denture teeth was reported by Goiato et al. and Campanha et al. after the exposure of teeth to a 4% CHX solution [28,29].

A long-term contact of PMMA denture resin with a number of chemical substances may also lead to an increase in solubility and sorption. In this study, the sorption levels of all the tested groups after 1 year of contact with CHX gels ranged from 20 to 24 µg /mm^3^, which is within the ISO 20795-1:2013 standard range for the sorption of resin after storage of 7 days (<32 µg/mm^3^). No evidence was found for the assumption that contact with CHX gels with ADSs influences the sorption levels of PMMA resin. The solubility levels of the specimens ranged from 6.74 (control group) to 6.84–7.91 µg/mm^3^. These results indicate that, after 1 year of conditioning, the solubility of the PMMA material in all the tested groups was greater than the value determined with 7 days of storage in distilled water (ISO 20795-1:2013 standard requirement <2 µg/mm^3^) [37]. The solubility of specimens stored with CHX gels was statistically higher than that of the control group, with the highest levels of solubility noted for the Curasept 1% group. None of the tested CHX gels caused a significant change in the sorption of PMMA denture resin; however, their presence increased the solubility of the resin.

A limitation of this study is that it was an in vitro work and cannot fully reproduce the complex oral cavity conditions. Moreover, the possible color alteration of denture material in correlation with pigments in food and drinks was not examined.

In clinical conditions, prolonged treatment with CHX is not advisable for patients with oral diseases and stomatopathies. However, many removable denture wearers may suffer from recurrent symptoms and hence use the CHX-containing disinfectant products for a longer period. The results of this study suggest that CHX used in the gel form at a concentration of 0.5% or 1% with ADSs, along with citric acid, caused only limited changes to color and mechanical properties of the PMMA denture base resin even when used for a prolonged time. Furthermore, the use of CHX-based products would not decrease the comfort and satisfaction of prosthetic patients.

## 5. Conclusions

The long-term use of disinfectant gels containing CHX and ADSs may cause a limited change in the color of PMMA resin and the mechanical properties of acrylic restorations. The results showed that CHX used in the gel form with ADSs at a concentration of 0.5% and the experimental gel containing 1% CHX with citric acid caused clinically acceptable changes to the color and mechanical properties of the PMMA denture base resin during the study period. These gels may be safely used by dental patients for oral hygiene regimen, even for prolonged periods of time. ADSs contained in these gels seem to be effective in preventing CHX discoloration and have no adverse influence on the mechanical properties of PMMA.

## Figures and Tables

**Table 1:** Composition of gels.

Gel	Composition
Experimental gel 1 1% CHX with citric acid 1% (self-manufactured)	Water, glycerol, hydroxyethyl cellulose, polyethylene glycol (PEG) 40 hydrogenated castor oil, 1% CHX digluconate, sodium citrate, citric acid monohydrate 1%
Experimental gel 2 1% CHX with 5% PVP (self-manufactured)	Water, glycerol, hydroxyethyl cellulose, PEG 40 hydrogenated castor oil, 1% CHX digluconate, sodium citrate, 5% polyvinylpyrrolidone (PVP)
Curasept 0.5% CHX (Curaden GmbH, Kriens, Switzerland)	Water, glycerol, xylitol, hydroxyethyl cellulose, 0.5% CHX digluconate, ascorbic acid, PEG 40 hydrogenated castor oil, sodium metabisulfite, aroma, methylparaben
Curasept 1% CHX (Curaden GmbH, Kriens, Switzerland)	Water, propylene glycol, hydroxyethyl cellulose, Poly(1-vinylpyrrolidone-co-Vinyl Acetate) (PVP/VA), PEG 40 hydrogenated castor oil, 1% CHX digluconate, sodium acetate, acetic acid, sodium metabisulfite, ascorbic acid

**Table 2:** Mean values ± standard deviation (SD) of color change (ΔE*)

Material	ΔE*
PMMA	1.49 ± 0.15 A
PMMA + gel 1 with citrate	2.6 ± 0.09 B
PMMA + gel 2 with PVP	3.6 ± 0.12 C
PMMA + Curasept 0.5%	2.4 ± 0.10 D
PMMA + Curasept 1%	3.67 ± 0.06 E

* Capital letters in columns denote statistically significant differences with *p* ≤ 0.05.

**Table 3:** Mean values ± SD of Brinell hardness

Material	Initial Brinell Hardness	Brinell Hardness after 1 year
PMMA	150.22 ± 1 A a	140.4 ± 1.4 A b
PMMA + gel 1 with citrate	150.22 ± 1 A a	143.3 ± 2.2 B a
PMMA + gel 2 with PVP	150.22 ± 1 A a	139.1 ± 2.2 C b
PMMA + Curasept 0.5%	150.22 ± 1 A a	143.1 ± 2.1 B a
PMMA + Curasept 1%	150.22 ± 1 A a	141.6 ± 1.53 D b

Lowercase letters in rows and capital letters in columns denote statistically significant differences with *p* ≤ 0.05.

**Table 4:** Mean values ± SD of sorption and solubility after 1 year of conditioning [µg/cm^3^].

Material	Sorption	Solubility
PMMA	21.9 ± 1.03	6.74 ± 0.95 *
PMMA + gel 1 with citrate	24.47 ± 1.5	6.84 ± 0.51 *
PMMA + gel 2 with PVP	24.34 ± 1.35	7.19 ± 0.78 *
PMMA + Curasept 0.5%	23.81 ± 1.03	7.34 ± 0.55 *
PMMA + Curasept 1%	22.83 ± 1.72	7.91 ± 0.75 *

* Statistically significant differences with *p* ≤ 0.05.
